# The role of the M-band myomesin proteins in muscle integrity and cardiac disease

**DOI:** 10.1186/s12929-022-00801-6

**Published:** 2022-03-07

**Authors:** Ekaterina P. Lamber, Pascale Guicheney, Nikos Pinotsis

**Affiliations:** 1 Department of Biomolecular Sciences, School of Life Sciences, Pharmacy and Chemistry, Faculty of Science, Engineering and Computing, Kingston University London, Penrhyn Road, Kingston upon Thames, KT1 2EE UK; 2 Institute of Translational Biomedicine, St. Petersburg University, St. Petersburg, 199034 Russia; 3 Research Unit on Cardiovascular and Metabolic Diseases, UMRS 1166, Sorbonne Université, Inserm, 75013 Paris, France; 4 Department of Biological Sciences, Institute of Structural and Molecular Biology, Birkbeck, Malet Street, London, WC1E 7HX UK

**Keywords:** Myomesin-1, Myomesin-2, M-protein, Myomesin-3, M-band, Sarcomere, Muscle, Cardiovascular disease

## Abstract

Transversal structural elements in cross-striated muscles, such as the M-band or the Z-disc, anchor and mechanically stabilize the contractile apparatus and its minimal unit—the sarcomere. The ability of proteins to target and interact with these structural sarcomeric elements is an inevitable necessity for the correct assembly and functionality of the myofibrillar apparatus. Specifically, the M-band is a well-recognized mechanical and signaling hub dealing with active forces during contraction, while impairment of its function leads to disease and death. Research on the M-band architecture is focusing on the assembly and interactions of the three major filamentous proteins in the region, mainly the three myomesin proteins including their embryonic heart (EH) isoform, titin and obscurin. These proteins form the basic filamentous network of the M-band, interacting with each other as also with additional proteins in the region that are involved in signaling, energetic or mechanosensitive processes. While myomesin-1, titin and obscurin are found in every muscle, the expression levels of myomesin-2 (also known as M-protein) and myomesin-3 are tissue specific: myomesin-2 is mainly expressed in the cardiac and fast skeletal muscles, while myomesin-3 is mainly expressed in intermediate muscles and specific regions of the cardiac muscle. Furthermore, EH-myomesin apart from its role during embryonic stages, is present in adults with specific cardiac diseases. The current work in structural, molecular, and cellular biology as well as in animal models, provides important details about the assembly of myomesin-1, obscurin and titin, the information however about the myomesin-2 and -3, such as their interactions, localization and structural details remain very limited. Remarkably, an increasing number of reports is linking all three myomesin proteins and particularly myomesin-2 to serious cardiovascular diseases suggesting that this protein family could be more important than originally thought. In this review we will focus on the myomesin protein family, the myomesin interactions and structural differences between isoforms and we will provide the most recent evidence why the structurally and biophysically unexplored myomesin-2 and myomesin-3 are emerging as hot targets for understanding muscle function and disease.

## Background

Muscle contraction is essential for life, covering involuntary functions such as heartbeat, as well as for our life quality, as it is necessary for most of our daily activities. To fulfil their role, muscles need to be able to withstand substantial mechanical forces while at the same time, they need to be able to stretch and reversibly return to their relaxing condition. Muscles can be divided into two major categories: the striated muscles, which are the cardiac and skeletal muscles, and the smooth ones. Striated muscles display organized and clearly distinguished contractile blocks mostly known as sarcomeres, while smooth muscles do not show any specific pattern. Interestingly, under physiological conditions, the cardiac muscle never gets tired contrary to the morphologically similar skeletal muscles. In that sense, the cardiac muscle represents a special kind of muscle and it is possibly the most important tissue in human body given that still in our days cardiovascular diseases remain the leading cause of death globally, representing the 32% of total deaths [1].

**Fig. 1:**
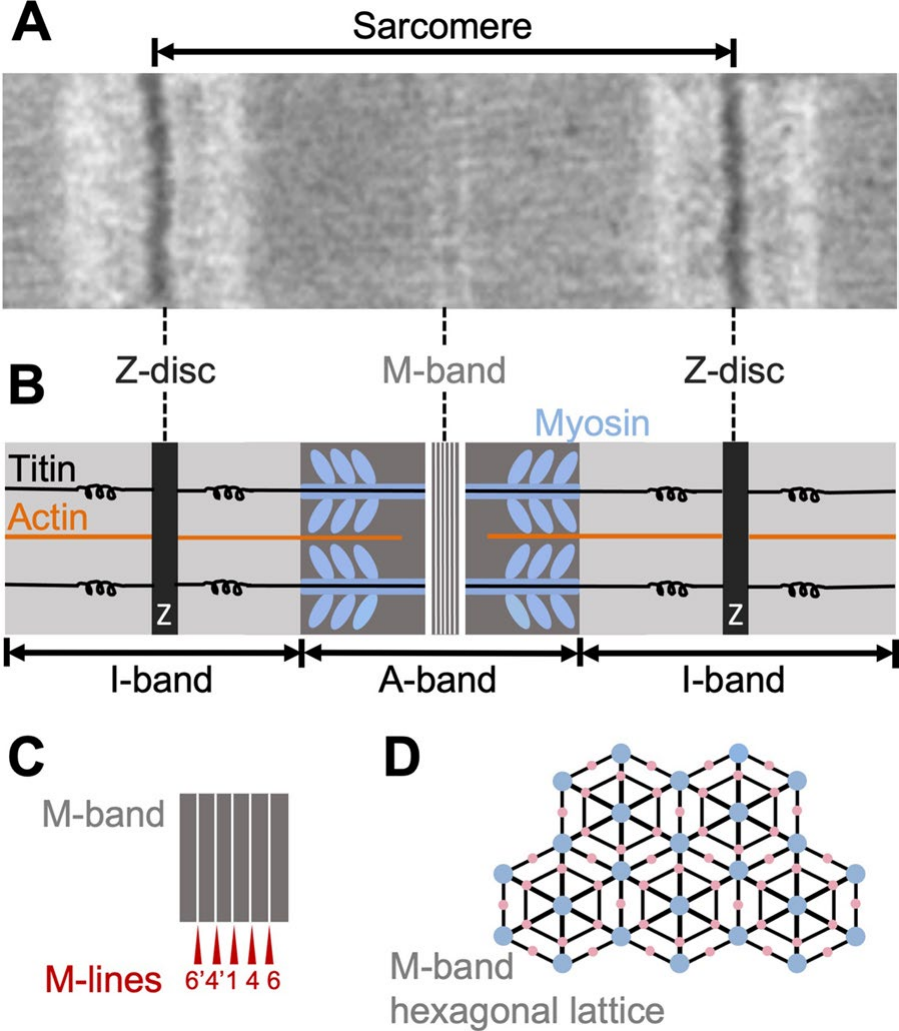
Sarcomere and M-band. **A** The muscle sarcomere as shown in an electron micrograph of longitudinally cut mouse heart muscle. **B** Schematic representation of the sarcomere shown in **A**. Myosin thick filaments are shown in blue, thin actin filaments in orange, sarcomeres borders (Z-discs) in black and the central zone (M band) in grey/white. The darker colors of Z-discs and A-band reflect on the striated appearance of sarcomere on electron micrographs. **C** A schematic representation of M band structure comprising of a set of M-lines (which appear as lighter lines on electron micrographs). **D** Transverse M-band lattice schematic representation based on electron microscopy sections. Blue circles indicate the positions of myosin filaments, small pink circles show the positions of linking filaments. The black lines indicate the positions of the linking material

Organized sarcomeric units form the basic contractile elements of skeletal and cardiac muscles [2, 3]. Sarcomeres are divided into specialized compartments that have different functions during the contraction. The sarcomere boundaries are defined by the Z-discs, which in electron micrographs appear denser than the rest of the sarcomere (Fig. 1A, B). The main purpose of Z-discs is to anchor the thin (or actin) filaments. The two main compartments within the sarcomere are the I-bands that contain only thin filaments, and the A-band that contains thin and thick (or myosin) filaments (Fig. 1A, B). In the central part of the sarcomere, known as the M-band, the myosin filaments interconnect and run antiparallel to both sides of the sarcomere (Fig. 1A, B). On the transversal sarcomere sections, the thick filaments form a regular hexagonal lattice (Fig. 1D). Specifically the Z-discs and M-bands, contain very dense networks of proteins that fulfill different functions: while the Z-discs are stiff and retain their structural integrity during contraction, the M-bands undergo huge conformational changes, and almost disappear before returning to their original regular pattern during muscle rest [4]. To support the contractile apparatus, the Z-discs and M-bands contain a complicated network of filamentous proteins, that are mainly comprised of arrays of immunoglobulin-like (**Ig**) and fibronectin type III (**Fn**) domains, as well as additional proteins involved in metabolic processes and signaling such as the α-actinin-2, a spectrin-like repeat dimeric protein that interconnects actin filaments in the Z-disc [5, 6]. These proteins are involved in a variety of interactions with each other, the thick and thin filaments, as also with adaptor and additional proteins that are implicated in signaling and various metabolic processes. The main protein here is titin which is also the largest single polypeptide found in nature, with a slack length of 1 μm and spanning the half sarcomere: from the Z-disc up to the central M-band (Fig. 1A, B). In addition to Ig and Fn domains, titin comprises several unstructured regions as well, such as the PEVK motifs in the I-band which provide elastic properties to the molecule [7]. Several additional proteins, localized in the M-band and Z-disc of the sarcomere, share similar domain layout to titin however, they have much smaller size. These include the myomesin family in the M-band, the filamins and the myopalladin in the Z-disc, the myosin binding protein C (MyBP-C) in the A-band and the obscurin proteins which are localized both in the Z-disc and the M-band [8, 9].

During the past decades the knowledge about the M-band has emerged from a low resolution structural model [10], to a compartment where the major components have specific structural roles and are involved in signaling processes [11], which possibly trigger in addition mechanosensitive responses [12]. Functionally, the M-band maintains the transverse hexagonal lattice of the myosin filaments and supports the centering and alignment of the A-band during muscle contraction and relaxation [13] (Fig. 1C, D). To achieve this task, the M-band contains few specialized proteins that together with their interaction partners form a dense protein network, comparable to that of the Z-discs in terms of overall complexity and functionality. Remarkably, in addition to obscurin more adaptor proteins such as the small ankyrin-1 and telethonin localize both in the Z-disc and M-band indicating common functions and links to the sarcomeric reticulum in both regions [8, 14].

In terms of architecture, electron micrographs of the M-band display vertical to the long sarcomere axis electron-dense lines which are known as M-lines (Fig. 1A–C). Five major M-lines can be resolved in striated muscle with a central to the sarcomere M1 line and two consecutive lines on each side at 22 nm intervals, known as M4/M4’ and M6/M6’ lines (Fig. 1C). While the density of the M4/M4’ lines is consistent in all muscle types [15], the density of the M1 and M6/M6’ lines varies depending on the muscle type. Additional lines are running parallel to the myosin filaments, and they are known as M-filaments [16].

The M-lines are mainly containing the ubiquitously expressed proteins, myomesin-1, the muscle isoform of creatine kinase (MM-CK) and the C-terminal part of titin comprising of 10 Ig domains diffused through large unstructured sequences into the entire M-band [15, 17]. Myomesin-1 and MM-CK appear to localize mainly on the M4/M4’ lines, while depending on the muscle type, additional myomesin isoforms are present in the M-band [17–19]. Although slow-twitch muscle fibers of mice deficient in MM-CK do not show any structural abnormalities, M-lines of longitudinal sections appear less intense. This suggests that MM-CK is not contributing to the formation of the M-band architecture [20]. In fast skeletal and cardiac muscles, the M1 line is more pronounced and is thought to be mainly composed of myomesin-2 [17]; while the third member of the myomesin family, myomesin-3, appears to be localized on the M6/M6’ lines of slow and intermediate speed skeletal muscles and cardiac muscles [18, 21].

Overall, the M-band architecture is the least studied part of the sarcomere mainly due to its dynamic properties [4, 22], given however the increasing number of publications that link this region to cardiovascular disease, it becomes apparent that there are still several unanswered questions related to its structure and function. This review will focus on the current knowledge of the myomesin protein family at a structural level and how this information contributes to a more complete M-band model. We will further examine the role of the different myomesin isogenes and isoforms and their possible positions in the M-band, which are related to different functions of the muscle as well as their emerging role in cardiomyopathies and other cardiovascular diseases. Finally, we will highlight the current open questions in the field that we expect to guide future research on the M-band.

### Myomesin protein family domain layout and sequence conservation

**Fig. 2:**
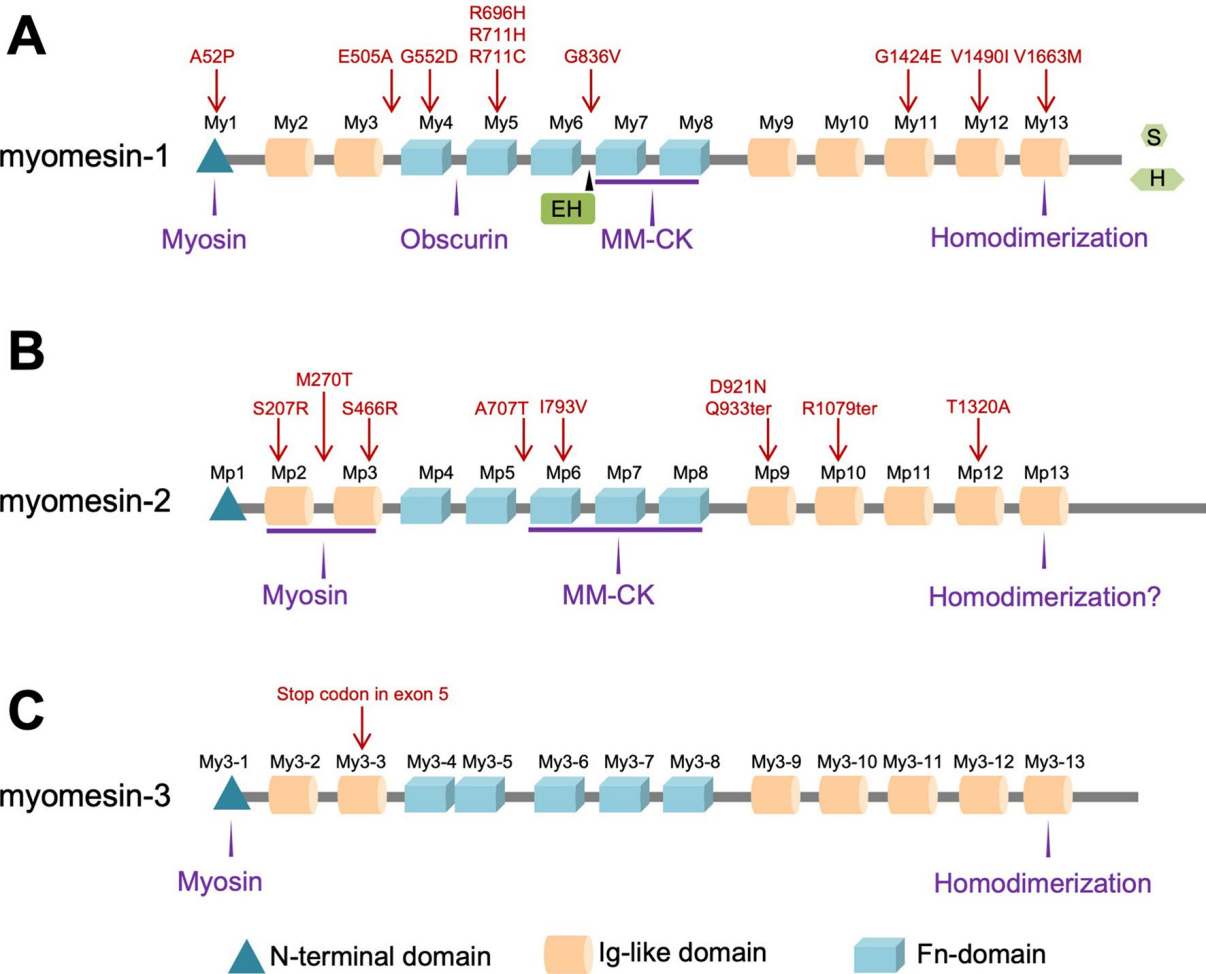
Domain layout of the myomesin proteins. **A** myomesin-1, EH-insertion, S and H splicing variants are shown in green **B** myomesin-2 **C** myomesin-3. Interacting partners are shown in purple, variants reported in Table 1 are shown in red above their approximate position in each molecule

All myomesin family proteins display the same domain layout of 13 distinct domains: a unique N-terminal domain followed by 2 Immunoglobulin-like (Ig) domains, 5 Fibronectin type III (Fn) domains and 5 Ig domains (referred in the text as My1 to My13 for myomesin-1, Mp1 to Mp13 for myomesin-2 and My3-1 to My3-13 for myomesin-3) (Fig. 2). They share a 50% sequence similarity with identity of about 40% mainly on the Ig and Fn domains. The major differences appear on myomesin-1, which is about 100 amino acids larger than the other two isoforms, are found at the N-terminal domain. Minor differences appear as well at the very C-terminus of the three proteins, suggesting that both termini should play a special role in the assembly and function of all proteins. Below we shall focus separately on each protein and their interactions.

### Myomesin-1: isoforms, structural insights, and interactions

Myomesin-1, first reported as a new M-band component in 1984, is currently the most studied protein among the myomesin family members [23]. It has two alternative splicing variants which are species dependent. The first variant is found in all species. It presents with a 96 amino-acid insertion between the Fn domains My6 and My7 known as the embryonic heart (EH)-sequence. The EH sequence is predicted to be intrinsically disordered [13, 24], thus this insertion in the center of the molecule should be adding elasticity to the molecule similar to the PEVK repeats in titin [25, 26]. EH-myomesin, is predominantly found in the embryonic heart of higher organisms [24, 27] and slow skeletal fibers in mice [13, 24, 28]. Apart from its obvious location in embryonic hearts, it has also been found in slow and extraocular muscle fibers of adult mice [26]. The second variant is located at the very C-terminus of the protein after domain My13 and to date it has been found only in avian muscles [13]. Compared to the approximately 20-residue sequence which is conserved at the C-terminus of all mammalian myomesin-1 sequences, the chicken heart myomesin contains an extended sequence of about 90 amino-acids. This is known as the H (H for Heart) splice variant. A shorter S-variant (S for skeletal) is found in the skeletal avian muscles [13, 29] which is quite similar in sequence and length to the mammalian myomesin-1 [29].

Unlike titin and obscurin which contain at least one kinase domain [8], myomesin proteins only contain domains that promote binding suggesting that myomesin regulation is achieved through binding partners. The N-terminal domain of myomesin-1 binds to myosin [30] via an interaction that is not yet completely elucidated, it is however essential for keeping the thick filaments in register [13]. Moreover, the N-terminal domain is about 120 amino-acids larger than the N-terminal domains of myomesin-2 and -3 and contains amino-acid repeats comprising a KQSTAS sequence which generates a very basic patch in the middle of the domain.

**Fig. 3:**
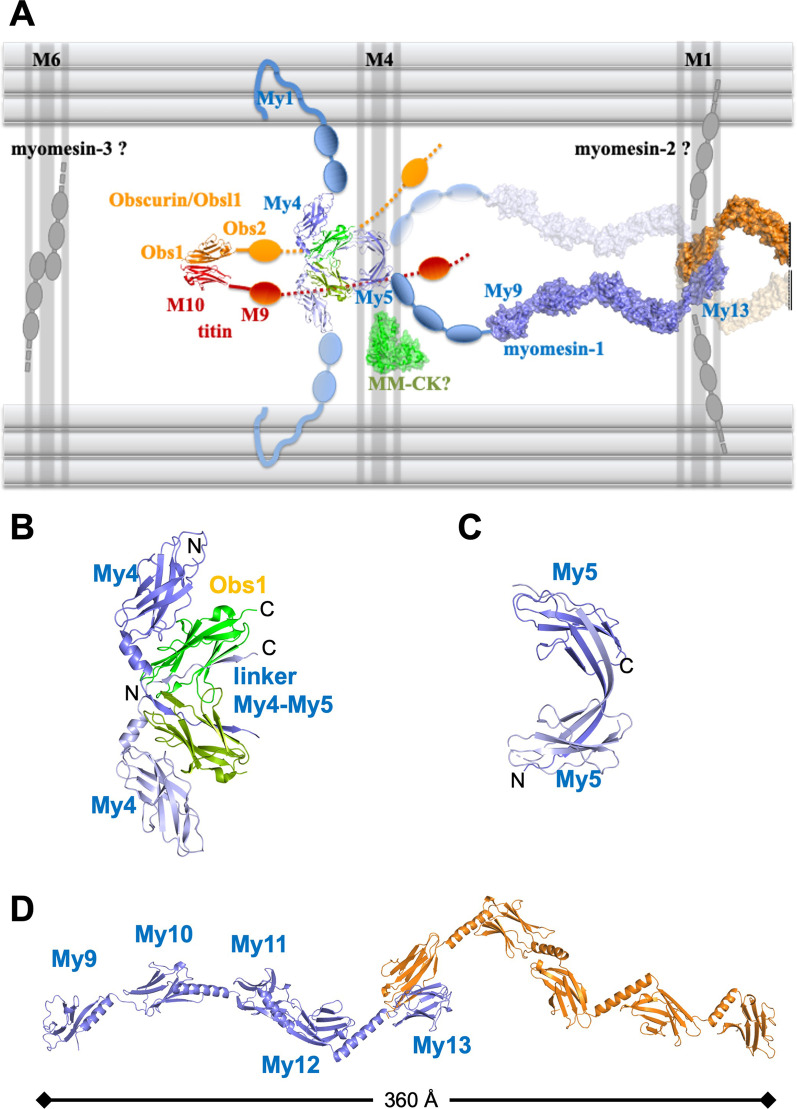
Structural organization of the major M-band filaments. **A** M-band organization and known structures. Determined crystal structures are shown in cartoon or in surface representation. Additional domains are shown as geometrical objects. Model not to scale. **B** Crystal structure of the myomesin-1 domains My4-(My4-My5)-linker with obscurin domain 1. Myomesin-1 domains are shown in blue and light blue and obscurin in green and light green. N and C-termini of the domains are indicated. **C** Crystal structure on myomesin dimerized domain My5 in blue and light blue. N and C-termini of the domains are indicated. **D** Crystal structure of the myomesin-1 domains My9-My13 as an antiparallel homodimer. Each chain is colored in blue and orange

The first interacting hub of myomesin-1 is located on the Fn domains My4-My6 that interact with titin Ig domain m4. Early reports using dot-blot assays, suggested that this interaction is abolished by phosphorylation of the myomesin amino-acid Ser618 (UniProtKB—P52179, corresponding to the Ser482 in the original work), located in the My4-My5 linker [30]. Nevertheless, more recent studies using pull-down and yeast two hybrid assays, do not confirm this interaction [31]. A subsequent study revealed molecular details on this region and specifically an interaction of the myomesin-1 My4-My5 linker with the Ig domain 3 of the proteins obscurin or obscurin like-1 (Obsl1) (Figs. 2A and 3A, B). This appears to be independent of Ser618 phosphorylation and this is also evident in the crystal structure where this residue is not involved on interface interactions (PDB ID 5FM5) [31, 32]. Interestingly, the myomesin domain My5 is not visible in the crystal structure of the myomesin My4-My5/obscurin complex, indicating that the My5 domain is rather flexible. The handshake arrangement of the myomesin and obscurin within the complex provides a stability and establishes a tight connection within the sarcomere as evidenced by a significant mechanical stability of the complex of about 135 pN force [32] (Fig. 3A, B). Further, a crystal structure of myomesin domain My5 (PDB ID 6ZVA) shows a dimerization of this domain which is compatible with the My4-My5/obscurin complex (Fig. 3A, C), although no publication is yet associated with this structure. The current structural picture of this region is enriched by a crystal structure of a relatively strong complex between the N-terminal Ig domain of obscurin or Obsl1 with the titin C-terminal Ig domain m10 [33, 34]. In conclusion, current data suggest that myomesin-1 forms a ternary complex with obscurin and titin at the M4/M4’ lines with multiple interaction points. Still, these interactions are not completely understood, not only in terms of regulation but also in terms of architecture: the latest data show dimerization interfaces both on domains My4 and My5, therefore it is plausible to assume that two filaments could run parallel towards the M1 line. More recent results using size exclusion chromatography and small angle X-ray scattering (SAXS) indicate a dynamic concentration dependent conformation of the My4-My5/Obscurin complex [32] which could be compatible with the aforementioned dimeric structure of domain My5 (PDB ID 6ZVA). The model of two myomesin filaments running parallel on each side of the M1 line can be further supported by a weak second dimerization interface that has been observed in all crystal structures containing the myomesin domain My13 (see below).

In the same proximity of the M4/M4’ lines myomesin is also involved in cellular energetics. Specifically the domains My7-My8 appear to interact with the muscle-type creatine kinase (MM-CK) [35]. Four lysine residues on the MM-CK isoenzyme are responsible for this interaction, which is dynamic and pH dependent based on the energy state of the muscle. Although the mechanism of creatine kinase function is common in various tissues such as in brain, involving replenishment of ATP from phosphocreatine in cytosol, the M-band interaction with myomesin-1 is the only one where the enzyme is in complex with a large modular protein [35]. Energy transfer in muscle sarcomeres is an essential requirement and apart from MM-CK, two additional metabolic enzymes have been found to also localize at the M4/M4’ lines, the adenylate-kinase (AK) and phosphofructokinase (PFK) [36]. These enzymes however interact indirectly through the LIM containing protein FHL-2/DRAL with the unstructured region of titin is2. The is2 region is located between the titin Ig domains m3 and m4 in the M-band. However, to date, there is no clear evidence the MM-CK/myomesin-1 interaction is following a mechanism similar to the one found for the AK and PFK enzymes [36, 37]. While related energetic mechanisms exist also in the Z-disc and the I-band [19, 36] the myomesin-1/MM-CK is unique, and its direct interaction is regulated by a very simple ionic-strength mechanism [38].

The five C-terminal domains of myomesin-1 belong to the Ig fold and, although this region of the molecule has not yet confirmed any intermolecular interactions, it has raised a lot of attention due to its homodimerization via domain 13 and its Ig α-helical linkers. Specifically, myomesin dimerizes via the domain My13, where two My13 domains interact through an antiparallel β-sheet assembly, suggesting that the two myomesin filaments should run to opposite directions from the central M1 line of the M-band [36, 39]. The hypothesis of an antiparallel elongated filament of two myomesin molecules is further supported by the linker between domains My12 and My13 which is found in an α-helical freestanding conformation [39]. An application of 30 pN force to this dimeric structure, resulted in a reversible elongation of the a-helical linker connecting domains My12 and My13, introducing for the first time a new concept of molecular elasticity in the region of Ig domains linked with standalone α-helices [40]. Additional crystal structures of the myomesin-1 domains My9-My13 combined with electron microscopy and X-ray solution scattering models unraveled a unique long 36 nm tail-to tail filament of 9 domains (4 on each side and a central dimerized My13) which are connected by α-helices (Fig. 3A, D) [22]. Application of forces smaller than 30 pN resulted in a reversible elongation of the molecule by about 2.5 times when compared to the original length which was explained as a reversible unfolding of the interconnecting α-helices between the Ig domains [22]. This mechanism is consistent with the previous M-band micrographs where under physiological tension conditions the band-associated thick filaments can move away from the sarcomeric central M-line by 0.1 μm or even more [4]. Overall, the reversible extension of the α-helical linkers provides a mechanism of the elasticity and allows myomesin to serve as a molecular spring.

**Fig. 4:**
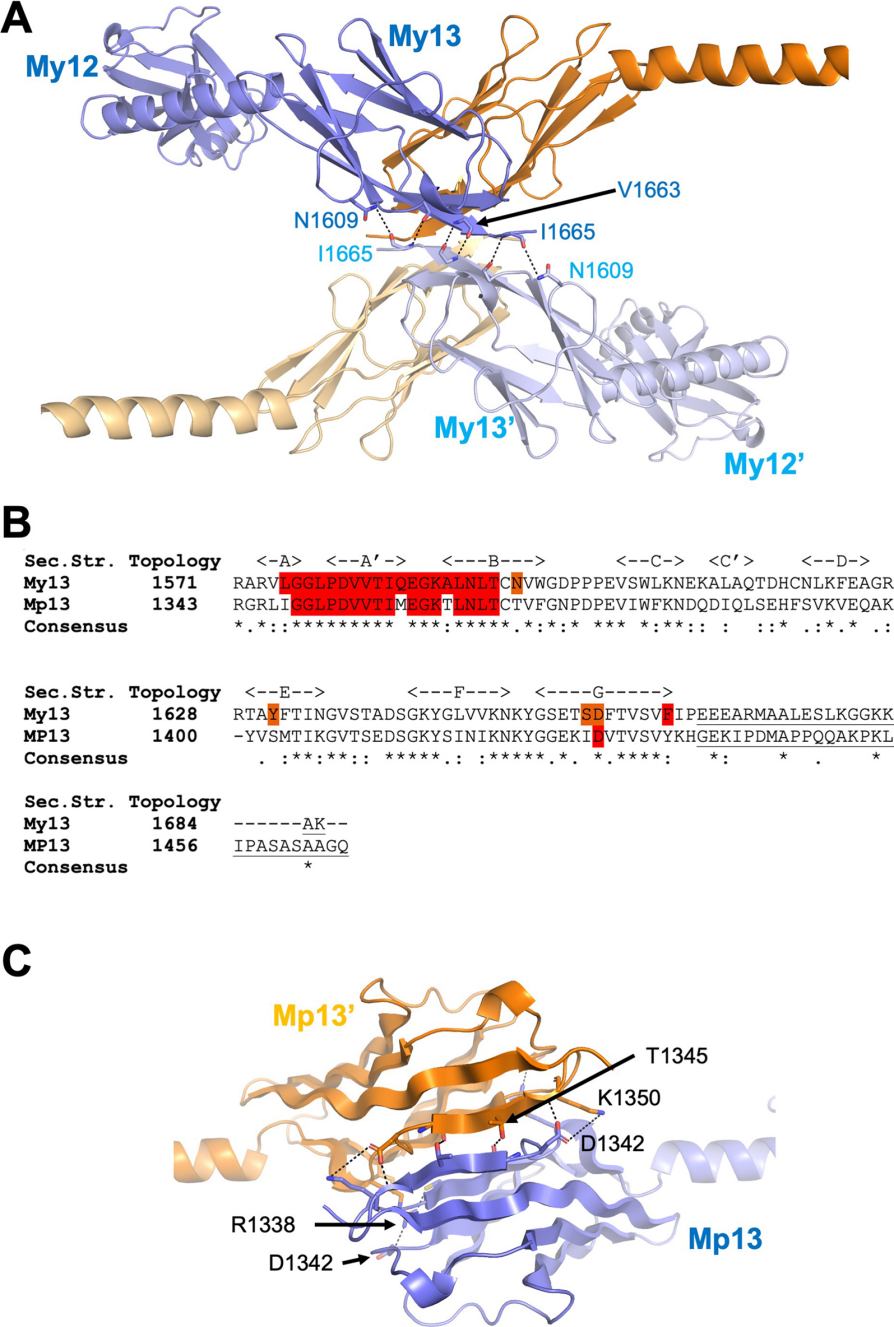
Domain 13 of myomesin-1 and -2. **A** Cartoon representation of the domain My13 as a tetrameric assembly highlighting the interactions of the second interface found in the crystals (PDB IDs 2R15 and 2Y25). Chain A (in blue) interacts with a space group symmetry related chain A from a neighbouring dimer. Hydrogen bonds and salt bridges are shown as dashed lines. The same interactions are detected in chain B molecules (orange and light orange). **B** Sequence alignment of domains My13 and Mp13. The amino-acids involved in the dimerization interface on My13 are highlighted in red as well as the conserved amino-acids of the interface on Mp13. The C-terminal amino acids of the proteins after domain 13 are underlined. **C** Cartoon representation of the Mp13 dimeric model. Hydrogen bonds and salt bridges are shown as dashed lines

Altogether, the current reported interactions and structural details render myomesin-1 as the central part of a multiprotein assembly important for the M-band architecture and function. There are still several unanswered questions and further work is required to address them focusing both in structural and cell biology. The most obvious discrepancy highlighted in this review, is the oligomerization state of the myomesin-1 filaments since a potential dimerization on the M4/M4’ lines would result a tetrameric assembly on the C-terminus. Could it be possible to have a myomesin filament bundling at the C-terminus able to support the observed complexes at the central part of the molecule? Although previous work on the domains My9-My13 show a single antiparallel dimer by negative stain EM and solution X-ray scattering [22, 39], the crystal structures revealed an additional conserved interface on domain My13 “hidden” under crystallographic symmetry (PDB IDs 2Y25, 2R15) (Fig. 4A), which can support two myomesin filaments running on each direction to the M4/M4’ lines (Fig. 3A). Possibly this interface is weaker, and it may need further support of yet unknown interactions, or from the existing interactions in the central Fn domains of myomesin-1. A clear answer on this could only be found in additional studies that can shed further light into the myomesin architecture and assembly.

### Myomesin-2: structural insights and interactions

Although myomesin-2 (also known as M-protein) was the first isoform discovered in 1974 [41], it has been less studied than myomesin-1, at least at structural and functional levels. Its domain layout is identical to the other family members; however, there are substantial differences in structure and function compared to the well-studied myomesin-1. To date there is only one isoform of myomesin-2 found with major differences in the protein termini: its N-terminus is much shorter than myomesin-1 [13, 42], and its C-terminus comprises a unique highly basic poly-proline sequence resembling no similarity to myomesin-1 (Fig. 4B).

Myomesin-2 is expressed mainly in fast skeletal muscles and in adult heart [13, 18, 43–45]. During embryonic development myomesin-2 is absent in favor of EH-myomesin both in human and mouse hearts [45]. A more recent study suggested that at least in mice the expression of myomesin-2 starts on the 12^th^ day of embryonic development [46]. However, this cannot lead to any conclusions about the exact time of its expression in human embryonic hearts, since the mouse myocardium generates much faster contractions, and therefore it has different dynamic requirements [47]. Furthermore, the myomesin-2 drosophila gene CG14964 is involved in a regulated interaction with myosin and controls the size of fly hearts [48].

As for myomesin-1, several interactions between myomesin-2 and other proteins of the M-band have been reported. At the N-terminus there is the expected interaction with myosin, however instead of the N-terminal domain reported for myomesin-1, myomesin-2 interacts through the Ig domains Mp2-Mp3 [49] (Fig. 2B). As mentioned above, the N-terminal domain of myomesin-2 is significantly shorter than myomesin-1, however with a very similar theoretical pI value of about 9.5. On the other side, the domains Mp2-Mp3 display a very high conservation to the domains My2-My3. Furthermore, in vitro experiments suggest a concentration dependent interaction between myomesin-2 and myosin, which can be abolished when the N-terminal unique domain gets phosphorylated on Ser76. [49]. Although there is no common pattern on how different myomesin proteins interact with myosin, it is clear that myomesin proteins occupy different compartments of the M-band [18, 30], therefore they should bind to different myosin regions. It is possible that the myosin interactions represent an additional safeguard that directs each myomesin protein to the correct position in the M-band.

Similar to myosin, the interaction of myomesin-2 with titin is unclear. Earlier biochemical work indicated a general interaction between titin and myomesin-2 [50]. Nevertheless, a later study showed that, when overexpressed, the myomesin-2 domains Mp4-Mp8 (corresponding to the myomesin-1 titin-binding domains), did not associate with myofibrils in C2C12 mouse myoblast cells, suggesting that if myomesin-2 binds to titin, this is not taking place through the central domains 4–8 as in the case of myomesin-1. In the same study, the domains Mp9-Mp13 recovered in myofibrils, however there was not sufficient evidence if the binding partner of these domains was titin or another structural protein of the M-band [49]. Still the complexity of interactions in eukaryotic M-bands combined with the variety of expression levels of myomesin-2 depending on the developmental stage or muscle type, clearly indicates that more work is required to confirm and understand how filamentous proteins in the M-band interact with each other. This is further supported by more recent work revealing molecular details of the interactions between titin, myomesin-1 and obscurin [31–34]. Undoubtedly, the linker connecting domains 4 and 5 in myomesin-1 interacts with the domain 3 of obscurin, while no interaction was observed between myomesin-2 and obscurin [31, 32]. Nevertheless, as we expect myomesin proteins to localize to different parts of the M-band, it is quite plausible that myomesin-2 could also interact with another filamentous yet unknown protein of the area in a similar fashion to myomesin-1.

Myomesin-2 domains Mp6-Mp8 interact with MM-CK, however they don’t bind in the same binding affinity as myomesin-1 [35] (Fig. 2). This interaction raises additional questions given that MM-CK is mainly found in the M4/M4’ lines [19], while the myomesin-2 Mp6-Mp8 domains were localized on the M1-line [17]. The current hypothesis implies that MM-CK displays a rather flexible and dynamic interaction within the M-band scaffold that can include more than one protein as interaction partner [35]. Definitely, the type of muscle cells used for MM-CK localization as well as the pH of the muscle play a role here [35], it is therefore plausible to assume that metabolic enzymes like MM-CK should be involved in additional interactions with scaffolding proteins. Myomesin-3 is also a potential candidate, and titin may be involved in an more direct interaction than the one previously reported [36].

The C-terminal part of myomesin-2 comprises five Ig domains and based on sequence comparison and secondary structure prediction, it very likely forms the same Ig-helix patterns as myomesin-1. Nevertheless, despite a similar domain pattern a main difference with myomesin-1 is that no dimerization of the protein has been observed on domain Mp13 by yeast two hybrid assay [18, 51]. This is rather surprising since sequence alignment reveals conserved residues in the dimerization interface (Fig. 4B). We further compared the AlphaFold [52] predicted model of the single domain Mp13 (model AF-P54296-F1) with the known structure of myomesin-1 domain My13 (PDB ID 2R15). Superposition of Mp13 onto the dimeric My13 showed no conflicting residues that could disrupt the dimerization interface; we also observed a high conservation of the hydrogen bonds and the salt bridges that are present in the My13 dimeric structure (Fig. 4B, C). Given the high sequence similarity between the two proteins, the most plausible scenario is that the unique C-terminus of myomesin-2 could be involved in a still unknown interaction, that either disrupts the interface in an auto-inhibitory fashion, or interacts with another yet unknown protein that regulates the dimerization of myomesin-2 or simply interferes with the previous experiments [11]. Clearly, additional investigations are required to explore a possible dimerization of myomesin-2, which has definitely important implications in the M-band architecture. Furthermore, as expected, no heterodimerization between myomesin-1 and myomesin-2 have been observed [18], a detail that not only suggests the differences in function, but also given the different interactions of the two proteins with myosin and obscurin, can act as a safeguard for the proper M-band assembly.

Given that the current data and the obvious comparisons with myomesin-1 are raising more questions than answering, it is more than clear that the structure and assembly of myomesin-2 in the M-band require much more attention than anticipated earlier. An additional reason for this is the fact that myomesin-2 is a protein specifically expressed in cardiac muscles (see also below).

### Myomesin-3: structural insights and interactions

Myomesin-3 is the latest member of the myomesin family that was discovered [18] and it the least studied. It appears to occupy the lines M6/M6’ of the M-band, and is found in skeletal fibers of intermediate speed [18, 53]. Its expression is also observed in adult hearts and specifically in left ventricle and its atrial appendance both in mice and humans [18, 21, 45]. Based on the current data, there is no myomesin-3 expression in embryonic heart tissues at any developmental stage [45].

Myomesin-3 interacts with myosin via its unique N-terminal region My3-1 (Fig. 2C). This region is similar in size (about 150 amino-acids) to myomesin-2 and has a less basic pI of about 8 and has no sequence similarity with any other N-terminal domain of the myomesin proteins apart from a predicted α-helical content. The other known confirmed interaction of myomesin-3 is its homo-dimerization via the domain My3-13 which in addition cannot be involved into a hetero-dimerization with the other members of the protein family [18]. Unlike myomesin-1 and -2 there is no linker between the domains My3-4 and My3-5, however the five C-terminal Ig domains are predicted to present the conserved Ig-helix motif found on myomesin-1 (AlphaFold model AF-Q5VTT5-F1). In summary, nowadays few is known about myomesin-3 function and interactions; as this protein is found in the cardiac muscle it should deserve more attention in future studies.

### The role of myomesin family in health and disease

The role of the myomesin proteins in health and disease is emerging through an increasing number of publications that link these proteins to various cardiomyopathies by mis-regulation of their expression levels.

The importance of the myomesin-1 and myomesin-2 in muscle tissues is highlighted by the deletion of *Mef2c*, encoding the transcription factor MEF2C, which directly binds to *MYOM1* and *MYOM2* promoters. In essence when *Mef2c* is absent, there is no myomesin-1 transcription, which in turn results severed M-bands and mice lethality during early embryonic states [54]. Similarly, the knockdown of myomasp/LRRC39, a protein involved in mechano-sensitive signaling pathways of the M-band, results in a significant downregulation of myomesin-1 and -2 proteins and a reduced contractile function that further triggers the expression of stress-responsive genes [55]. By generating a CRIPR/Cas9 edited human embryonic stem cell line *MYOM1*^−/−^ differentiated into cardiomyocytes, it has recently been shown, that myomesin-1 deficiency induces atrophic features and morphological abnormalities. In those cells, myocardial dysfunction was observed, possibly due calcium homeostasis disturbance [56].

Changes on the expression pattern of the myomesin proteins can be an indicator for cardiac diseases. Of particular interest here is the expression levels of EH-myomesin which has been associated with dilated cardiomyopathy (DCM) in mice and humans. Two different well-established DCM models, the cΔex3 β-catenin mice and the Muscle LIM Protein knockout (MLP-KO) mice, showed a consistent elevated EH-expression with a parallel downregulation of myomesin-2. While in the first model (cΔex3 β-catenin mine), the myomesin-3 levels remained unchanged, in the second model (MLP-KO mice) myomesin-3 was upregulated suggesting a discrimination between different DCM mouse models [45]. In humans, in DCM cardiac tissues an EH-myomesin elevation was reported with a normal expression of myomesin-2 and -3. A possible explanation for this discrepancy could be linked to the much higher contraction speed of mouse heart which require further “adjustments” on the myomesin expression levels. Still, the EH-myomesin upregulation of DCM human hearts displayed a significant 41-fold increase compared to control and hearts with hypertrophic cardiomyopathy (HCM) groups. This kind of expression regulation is strikingly similar to the one found in healthy embryonic M-bands, characterized by high EH-myomesin and low myomesin-2 expression levels and is in line with up-regulation of compliant titin isoforms in human DCM. These findings show an impressive adaptation of the heart in special conditions: heart dilatation simply requires overstretched conditions, and the upregulation of a more elastic version of the myomesin proteins seems a simple and reasonable solution for contraction regulation [13, 26, 45].

Another link of myomesin-1 to sarcomere hypertrophy is through the SUMOylation of myomesin-1 from the myofibrillogenesis regulator-1 (MR1). Specifically, MR1 overexpression showed an increased nuclear to cytoplasmic translocation of myomesin-1 which mimics SUMO1 overexpression. As a result, myomesin-1 mediates increased sarcomere organization preceding hypertrophy [57, 58].

In another study, myomesin-2 has been reported to interact with the ryanodine receptor type 1 (RYR1). This interaction was identified using a double hybrid screening of a cDNA bank prepared from human skeletal muscle and confirmed by a Duolink^®^ proximity ligation assay [59]. Myomesin-2 may also interfere in cardiomyocytes with the ryanodine receptor type 2 (RYR2), a protein essential for Ca^2+^ release in heart as well as in cardiac arrhythmias. Interestingly, a functional link between myomesin-1 and Ca^2+^ regulation has recently been described [56].

Finally, myomesin appears to have an aberrant regulation in myotonic dystrophy type 1 (DM1), which affects several tissues including heart. Specifically, DM1 muscles show embryonic splicing abnormalities in numerous genes, and is has been suggested that the increase of the *MYOM1* exon17a inclusion which encodes the EH-domain of myomesin, is one of these DM1 abnormalities [60]. Over-expression of the RNA binding “muscleblind-like” (MBNL) family proteins, decreased the inclusion of exon 17a, thus suggested a correlation between DM1 and EH-myomesin, without however specifying a role of myomesin in DM1 [60].

### Myomesin variants

In terms of pathogenicity, a continuously increasing number of single point variants is emerging over the past years to all myomesin proteins that can potentially link them to disease (Table 1, Fig. 2). Nevertheless, due to the lack of sufficient knowledge and functional studies of myomesin-1 and myomesin-2 in myocardial cells, their biological functions and their role in heart diseases have not yet been clarified.

**Table 1:** Selection of *MYOM1* and *MYOM2* variants: Selection of published variants identified in patients with hypertrophic (HCM), dilated (DCM) or restrictive (RCM) cardiomyopathy, embryonic lethality (EL), sudden unexplained death in young (SUDY), sudden cardiac death (SCD), arthrogryposis (Arthr) and Tetralogy of Fallot (TOF)

Gene	GRCh37/hg19	HGVSc	HGVSp	Zygosity	Mutation taster	Varsome	HGMD disease mutation	CADD	GnomAD v2.1.1	MAF	Associated disease	References
MYOM1	18-3215068-C-G	c.154G > C	p.A52P	Htz	Polym	VUS	CM188378	22.2			SUDY	[76]
MYOM1	18-3155074-T-G	c.1514A > C	p.E505A	Htz	DC	VUS	CM1715431	24.7	3/277564	0.00001081	HCM	[70]
MYOM1	18-3151880-C-T	c.1655G > A	p.G552D	Htz	DC	VUS	CM1620054	27.4			HCM	[70]
MYOM1	18-3135667-C-T	c.2087G > A	p.R696H	Htz	DC	VUS	CM1620045	28.8	57/280526	0.0002032	HCM	[70]
MYOM1	18-3135622-C-T	c.2132G > A	p.R711H	Htz	DC	VUS	CM168783	32.0	22/280536	0.00007842	HCM	[77]
MYOM1	18-3135623-G-A	c.2131C > T	p.R711C	Htz	DC	VUS	CM168778	29.4	9/249116	0.00003613	HCM	[77]
MYOM1	18-3129517-C-A	c.2507G > T	p.G836V	Htz	DC	LB	CM173090	33.0	9/242760	0.00003707	HCM	[71]
MYOM1	18-3085111-C-T	c.4271G > A	p.G1424E	Htz	DC	VUS	CM1611742	32.0	10/270868	0.00003692	RCM	[78]
MYOM1	18-3083803-C-T	c.4468G > A	p.V1490I	Htz	DC	VUS,B	CM110808	23.2	6/188284	0.00003187	HCM	[65]
MYOM1	18-3067331-C-T	c.4987G > A	p.V1663M	Hmz	DC	VUS, LP	CM155525	28.7	2/249186	0.000004013	EL	[62]
MYOM2	8-2007334-C-G	c.621C > G	p.S207R	Hmz	DC	LB		22.8	16/282774	0.00005658	Arthr	[63]
MYOM2	8-2020440-T-C	c.809T > C	p.M270T	Htz	Polym	LB		10.9			HCM	[48]
MYOM2	8-2026950-C-G	c.1398C > G	p.S466R	Htz	DC	VUS		23.4	157/282714	0.0005553	HCM	[48]
MYOM2	8-2041912-G-A	c.2119G > A	p.A707T	Htz	DC	LB	CM147958	22.4	25/282664	0.00008844	TOF	[46]
MYOM2	8-2046750-A-G	c.2377A > G	p.I793V	Hmz	Polym	VUS		1.04			HCM	[48]
MYOM2	8-2054058-G-A	c.2761G > A	p.D921N	Htz	DC	VUS	CM1515391	24.6	26/282488	0.00009204	SCD	[72]
MYOM2	8-2054094-C-T	c.2797C > T	p.Q933ter	Hmz	DC	VUS		47.0	26/251264	0.00010347	EL	[63]
MYOM2	8-2063806-C-T	c.3235C > T	p.R1079ter	Htz	DC	VUS		40.0	7/251336	0.00002785	HCM	[48]
MYOM2	8-2088749-A-G	c.3904A > G	p.T1302A	Htz	DC	LB	CM147960	16.3	93/282810	0.0003288	TOF, DCM	(46)

Variants homozygous in GnomAD large populations were not introduced

*Htz* heterozygous, *Hmz* homozygous, *DC* disease causing, *VUS* variant of unknown significance, *LB,B* likely benign, benign, *MAF* minor allele frequency from total exome and genome from GnomAD v2.1.1

No variant was reported as pathological in the clinical databases such as OMIM (https://www.omim.org) or ClinVar (https://www.ncbi.nlm.nih.gov/clinvar/), they are considered as likely benign or variants of unknown significance (VUS). The encoding genes *MYOM1*, *MYOM2* and *MYOM3* have more functional genetic variations than expected based on the apparently neutral variations found in the genes (https://gnomad.broadinstitute.org). They are classified as tolerant to functional genetic variations in contrast to most of the genes responsible for Mendelian diseases. Since myomesins are large proteins with repetitive elements, it is not surprising that numerous benign or of unknown significance variants were reported in population databases from exome or genome sequencing, as in the case for titin gene, *TTN* [61]. Many of them are probably frequent or rare benign variants, as well as errors of sequencing due to the homology of the repeated domains.

Nevertheless, due to the importance of variants in diverse sarcomeric proteins leading to skeletal and cardiac muscles, we can hypothesize that M-band components could also contribute to the genetics of these muscle disorders, possibly as major effectors in some very rare cases or as oligogenic components or susceptibility genes in others.

First, some homozygous variants have been reported in *MYOM1* and *MYOM2* genes leading to early lethality. In consanguineous families from Saudi Arabia in which embryonic lethality segregates as a recessive Mendelian phenotype, autozygosity mapping and whole-exome sequencing revealed *MYOM1* as a potential embryonic lethal gene [62]. They identified a homozygous missense variant, p.Val1663Met, located in My13 and although this variant is not involved in any reported interaction, Val1663 is the central residue for a possible second dimerization interface on My13 as presented above (Fig. 4A). Besides, in unrelated consanguineous Turkish families, two homozygous variants have been identified in *MYOM2* and reported as potential causatives for arthrogryposis [63]. A missense variant, p.Ser207Arg, was found in a young child with arthrogryposis at birth. In the second case, a fetus terminated at 20 weeks of gestation was a carrier of the homozygous nonsense variant, p.Gln933ter and presented as also additional abnormalities including cardiac ones [63]. Noteworthy, no or a very small number of homozygous loss of function (LoF) variants have been identified in large screened populations. At functional level, Pehlivan et al. suggest that these variants may affect the interaction with dysferlin and this appears as a plausible scenario for the p.Gln933ter variant [64]. On the other side, the position of the p.Ser207Arg variant, based on sequence analysis and a recently predicted AlphaFold model, is located on the domain Mp2 which has been reported to interact with myosin [49]. It is therefore likely that the p.Ser207Arg variant may have a direct impact on the sarcomere integrity by affecting the myosin-myomesin-2 assembly.

Secondly, several missense variants have been described in cohorts of patients with hypertrophic (HCM), dilated (DCM) or restrictive (RCM) cardiomyopathies, often late-onset pathologies considered with 50% of genetic components and oligogenic components. The first identified variant, p.Val1490Ile, in *MYOM1* screened as a candidate gene, was transmitted from a mother to her two sons, all affected by HCM [65]. This change occurred on the Ig domain My12 and it was reported to affect the protein properties [65]. Given however the available structural information on the domain (PDB IDs 2Y25 and 2R15), we can observe no involvement of the Val1490 to the dimerization of the protein or any interaction with the connecting My12-My13 α-helix. In addition, we can only assume minimal effects on the biophysical properties of domain My12 since Val1490 targets the core of the domain on a position where Methionine is also a possible substitution based on the sequence conservation of β-barrel domains [22, 66–68]. Nevertheless, we cannot exclude this variant to be affecting a novel yet unknown interaction or protein modification.

Reported sporadic variants are also given in Table 1 with, for most of them, a Combined Annotation Dependent Depletion (CADD) score compatible with pathogenicity (> 15) [69]. Noteworthy, only *MYOM1* has been included as a candidate gene in most of the genetic screening of patients with cardiomyopathy or sudden unexplained death (SUD). In several studies, *MYOM1* variants have been associated with variants in the major HCM genes, suggesting a limited—if any—functional effect [70, 71]. Some heterozygous *MYOM2* variants were also reported in patients with Tetralogy of Fallot, but we excluded several of them since they were present at homozygous state in the gnomAD screened populations [46].

Interestingly, a very rare variant, p.Asp921Asn in *MYOM2* which is located on the Ig domain Mp9, was reported in a patient with sudden cardiac death [72].

Lastly, a nonsense variant in MYOM3, p.Glu144Ter, was reported in DCM patients [21]. This variant in linkage disequilibrium with a missense variant, rs149105212, is specific to Asian populations. This is surprising since myomesin-3 is poorly expressed in heart and preferentially in intermediate skeletal muscle fibers (Agarkova et al. 2003, Gautel Djinovic-Carugo 2016 [44]; Schoenauer et al. 2005 [26]). The authors suggested that it may increase the risk of DCM, but this has to be confirmed in larger populations.

Overall, clearly variants that can affect key residues in domains responsible for cross-linking with other proteins of the M-band such as, myosin, titin or obscurin, or proteins involved in Ca^2+^ homeostasis may destabilize the sarcomere inducing myofibrillar disarray. By altering contractility and modifying the Ca^2+^ pathways, they could contribute to the development of a cardiomyopathy or the development of ventricular fibrillation leading to sudden death. Biochemical studies should be developed to better identify binding partners of the myomesin proteins as well as functional studies to determine the contribution of myomesin variations in human pathologies.

## Conclusions

The M-band architecture and function were always a major challenge for structural and cell biologists where early work was focusing on the ultrastructural organization of the region [10, 15, 16, 38, 73, 74]. Contrary to other compartments of the muscle sarcomere, the M-band is the most dynamic and displays huge conformational changes [4]. This unique behavior is limiting the electron microscopy studies only to tissues that have organized M-bands. Such tissues are usually limited in few species and are prepared under very specific conditions that may not be very representative of normal M-bands in mice or humans [10, 73]. In parallel, the discovery of the major molecular components of the M-band like the M-protein (myomesin-2), myomesin-1, MM-CK, titin and then obscurin [19, 30, 41, 43, 75] resulted an increased interest for the architectural and functional composition of the region, initially with epitope labeling and biochemical characterization of specific components [17, 38, 51] and later by high resolution structural biology approaches [22, 39].

While the M-band has been studied for decades, there are still unanswered questions related to M-band puzzle. Currently, we have a more detailed overview of the M-band architecture, having elucidated several inter and intramolecular interactions and having a good understanding of the structural details and conservation of the β-barrel domains that constitute the building blocks of the major filament proteins in the region. We are still missing important details about the components that are supporting the myosin hexagonal lattice [44] while the structural details on the other two proteins of the myomesin family are remaining elusive despite the high degree of similarity between the three isogenes. Since each myomesin protein localizes to a different compartment of the M-band, we can expect different interaction partners and clearly there are several open questions that need to be answered. For example, do myomesin-2 and myomesin-3 interact with titin and obscurin? And if they do interact, which are the interacting domains? Are there any other filament proteins in the M-band that we are missing? Does myomesin-3 interact with MM-CK and, if it is the case, can we identify this enzyme in the M6/M6’ lines? What happens when a myomesin protein overexpresses in the M-band and what is the actual structural role of EH-myomesin in health and disease? How does the regulation of expression for myomesin proteins work and is this compensation beneficial? Future research studies able to answer such questions thus adding more pieces to the M-band puzzle, will help us to elucidate the mechanisms underlying the ability of M-band to withstand the force and to be able to stretch. Furthermore, fine details on the M-band architecture will also contribute to understanding the impact of disease-associated variants that emerging in all myomesin proteins.

In summary, M-band, known as the most dynamic part of the muscle, shows extraordinary elasticity and large conformational changes during contraction, therefore even minor details in protein composition may eventually create severe functional defects. Consequently, we need a combined approach of techniques including structural and cellular biology together with biophysical methods, genetics and animal models, in order to attribute disease variants to specific phenotypes that are compatible with the architecture and the signaling/interacting processes of the M-band.

## Data Availability

The datasets analysed during the current study are publicly available from the protein data bank (https://www.rcsb.org/). Figures showing structures were prepared using the PyMOL Molecular Graphics System, Version 2.5.0 Schrödinger, LLC.

## Abbreviations

MMCK: Muscle isoform of creatine kinase
Ig: Immunoglobulin-like domain
Fn: Fibronectin type III domain
DCM: Dilated cardiomyopathy
HCM: Hypertrophic cardiomyopathy
MLP-KO: Muscle LIM protein knockout
MR1: Myofibrillogenesis regulator-1
SCD: Sudden cardiac death
